# An Online Pain Education Program for Working Adults: Pilot Randomized Controlled Trial

**DOI:** 10.2196/15071

**Published:** 2020-01-14

**Authors:** Yajie Li, Mun Yee Mimi Tse

**Affiliations:** 1 School of Nursing The Hong Kong Polytechnic University Hung Hom, Kowloon Hong Kong

**Keywords:** pain, online education, WeChat, working adults

## Abstract

**Background:**

Pain is a common public health concern, and the pain situation among the general population is serious in mainland China. Working adults commonly experience pain because of long sitting times, a lack of free time, and exercise. A lack of pain-related knowledge is also a significant factor. Educational and therapeutic programs delivered online were used more often in Western countries, and accessible programs in China are limited, especially for pain management. Therefore, we carried out an online pain education program for working adults to self-manage pain. The program was delivered through WeChat, a popular and secure social media with a large population base in China.

**Objective:**

This study aimed to (1) provide pain-related knowledge and self-relief strategies, (2) help participants reduce pain and improve pain-related emotional well-being, and (3) explore participants’ learning performance and the acceptability of the online pain education program.

**Methods:**

This was a randomized controlled trial. Chinese adults aged between 16 and 60 years with full-time employment, with pain in the past 6 months, and without any mental illness were recruited using snowball sampling through the internet and were randomly allocated to an experimental group and a control group in 1:1 ratio after the baseline assessment. The 4-week educational program that included basic knowledge of pain, pharmacological and nonpharmacological treatments, and related resources was provided only to the experimental group. Outcomes of pain, depression, anxiety, stress, and pain self-efficacy were measured at baseline (T0), posttreatment (T1), and 1-month follow-up (T2). Participants’ acceptability and satisfaction were explored after completing the educational program.

**Results:**

In total, 95 eligible participants joined in the program: 47 in the experimental group and 48 in the control group. Neck and shoulder, head, and back were most commonly reported pain sites with high pain scores. Pain intensity and interference of the experimental group were significantly reduced after the educational program. Depression, anxiety, and stress clinically improved and pain self-efficacy improved after the educational program. The difference in depression, anxiety, stress, and pain self-efficacy within a group or between groups was not statistically significant; however, clinical improvements were demonstrated. A significant correlation between dosage of the intervention and pain intensity and depression was demonstrated. After completing the educational program, more than half of the participants showed acceptance of and satisfaction with the program, and they were willing to recommend the program to others.

**Conclusions:**

Our findings highlight the significant potential of this online education program in the treatment of pain.

**Trial Registration:**

ClinicalTrials.gov NCT03952910; https://clinicaltrials.gov/ct2/show/NCT03952910

## Introduction

Pain is a common and major public health concern [1-3] with a high negative impact on different aspects of the affected individual’s quality of life [4-8]. Pain prevalence in developing countries was reported to be approximately 40% among the general population [9]. In Asia, the prevalence of pain in adults ranges from 7.1% to 61% [10]. Studies from China showed a serious pain situation where the estimation of pain prevalence was approxiamately 40% [11-13]. The pain situation of working populations should be taken into consideration, as long sitting time and computer-facing time can lead to discomfort of the body, especially in the neck, shoulders, and back [14].

Many face-to-face pain management programs have been carried out to control pain and reduce its negative impact [5,15-17]. However, the internet has been used as an innovative approach to deliver these programs using the same principles, providing same evidence-based treatments, and teaching the same skills as those delivered face to face [1,18,19]. The internet offers a viable way to deliver self-management support for assisting patients in managing a wide variety of conditions and has the potential to overcome many barriers of the face-to-face approach. One of the obvious benefits is availability of the programs; participants can access them at their convenience and pace, which may provide better control of their situation and yield a greater outcome [20]. As the use of the internet and social networking increased, the increase in health care use via these modes was inevitable [21]. Increasing evidence shows that internet-delivered educational and therapeutic treatments have high accessibility and acceptability [22].

The internet is widely used in China. The China Internet Network Information Center reported that more than 55% of the Chinese population was using the internet by December 2017; among these internet users, 97.5% were using a mobile phone [23]. WeChat is a popular free mobile app for communication and accessing the internet. WeChat attracted more than 900 million active users as of September 2017 [24]. In recent years, subscription, as a new plug-in in WeChat, is a new means to propagate information under a safe condition and is becoming increasingly popular [25,26].

Although the use of online programs to help people with pain is a logical way to overcome many existing barriers, there is limited research in China focusing on illustrating the effectiveness of such programs among working adults. To the best of our knowledge, only one study used the internet to deliver a pain management program for teenage girls to self-manage dysmenorrhea in China and proved its effectiveness [27]. Therefore, we conducted an online pain education program through WeChat for self-management of pain among working adults. The study evaluated the effectiveness of the online pain education program in reducing pain and improving pain-related emotional well-being. We also evaluated participants’ learning performance and acceptability.

## Methods

### Study Design

The study was designed as a randomized controlled trial that examined the effectiveness of a 4-week online pain education program. The study was approved by the ethical committee of The Hong Kong Polytechnic University (ref. HSEARS20180519002). Data were collected from September 2018 to March 2019.

### Participants

An online poster explaining the details with a quick response (QR) code for the app was designed and distributed in WeChat to attract participants. Individuals who were interested in participating could scan the QR code to register in the study. A total of 152 people applied to join in the program. The application process involved completing an online questionnaire to screen eligibility. Eligible participants were then randomly allocated to one of the two groups: (1) the group that received the pain education program (experimental group) or (2) the group that received only simple material (control group). To minimize the potential of study bias, randomization was performed using an online randomizer [28] with 1:1 ratio after the registration period by a person who was not involved in this study.

**Figure 1:**
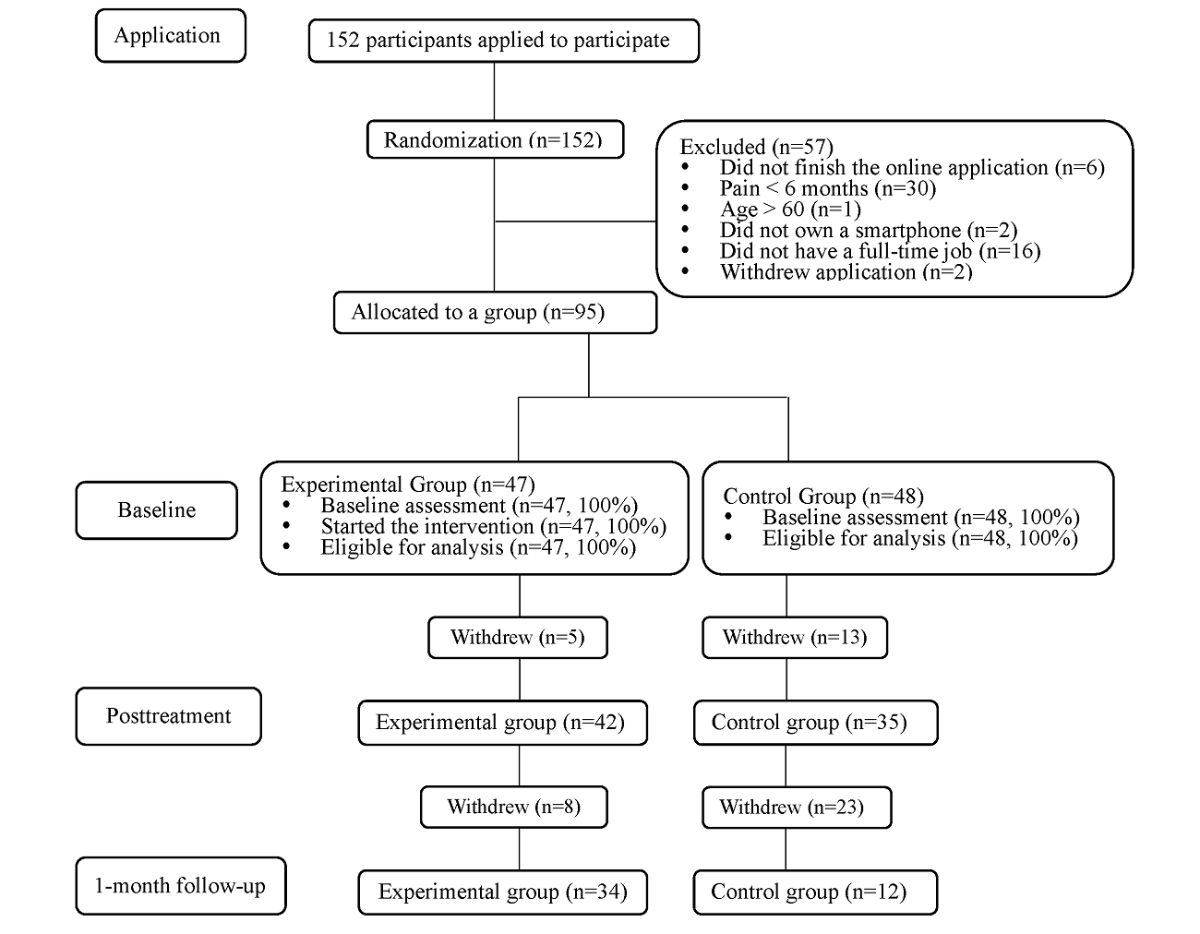
Consolidated Standards of Reporting Trials map.

Participants were required to fulfill the following criteria: (1) presence of noncancer pain in the past 6 months, with a pain score of at least 2 when assessed using an 11-point scale; (2) age between 16 and 60 years; (3) full-time employment; (4) ability to understand Chinese; and (5) ownership of a smartphone to access the internet. Those who had mental disorders, drug addiction problem, or further treatments planned were excluded from this study. Of the 152 people registered, 95 fulfilled the criteria: 47 were allocated to the experimental group, and 48 were allocated to the control group. The Consolidated Standards of Reporting Trials map for this study is shown in Figure 1.

### Ethics, Consent, and Permissions

Before the eligibility assessment, individuals’ consent was obtained electronically, emphasizing that participation was voluntary and remunerative. It was clarified that withdrawal from the study was accepted at any stage. Participants were also informed that all their personal information would remain confidential.

### Experimental Group Versus Control Group

#### Experimental Group

The intervention provided for the experimental group in this study was the online pain education program, which encouraged participants to learn and practice the knowledge and skills introduced in the program. An overview of the program is presented in Multimedia Appendix 1.

The program content including basic knowledge of pain, physical and psychological impact of pain, pharmacological and nonpharmacological treatments, and relevant resources were uploaded to the subscription in a short article format at the beginning of the program. Each article took approximately 3-5 min to read without order restriction. In addition, participants could interact with each other in the program. At the end of each article, three to four multiple-choice questions (MCQs) were asked depending on the article, and all the participants were required to answer the questions. Unlimited attempts were allowed for the MCQs, and log in with WeChat ID was required. Correct answers were provided and would be available after completing the MCQs. The score of each participant was used to evaluate an individual’s learning performance. In addition, a WeChat group was created for the participants in the experimental group, and they were encouraged to discuss issues related to the pain education program and share their learning experience. Materials were always accessible during the available period. Regular reminders on a weekly basis were sent to the participants through WeChat.

A total of five experts were invited to assess the content validity using content validity index, including two registered nurses, two pain specialists, and one expert in traditional Chinese medicine. The result of the content validity index was 0.95, which indicated that the program was validated [29]. Test-retest reliability was performed by 10 people 2 weeks apart, and the results ranged from 0.82 to 0.96, suggesting that the content was reliable [30].

#### Control Group

Brief (one page) material related to pain (that was obtained from an online leaflet open for public) from a grade A tertiary hospital in China [31] was given to the control group in WeChat at the beginning of the program. These participants were required to read the material whenever possible during the 4-week study period.

### Outcome Measures

Outcome measures were administered online at three time points: (1) baseline (T0): after randomization and before starting the education program, (2) posttreatment (T1): right after the experimental group finished the program, and (3) follow-up (T2): 1 month after finishing the program. A battery of well-designed questionnaires was used for outcome measures. The primary and secondary measures were administered at T0, T1, and T2. Participants’ learning performance, satisfaction, and acceptability assessment were administered after the intervention (ie, T1). The questionnaires were uploaded in WeChat at three outcome measurement points. To facilitate a high completion rate of the assessment, a reminder message was sent to the participants individually.

### Primary Outcome

#### Brief Pain Inventory - Chinese Version

The Brief Pain Inventory (BPI) is a brief, self-administered questionnaire, which is designed to measure the pain intensity and impairment caused by pain. It consists of four questions related to pain severity and seven questions related to pain interference. The pain interference items focus on general activities, mood, walking ability, work, relationship with others, sleep, and enjoyment of life. A previous study showed that the BPI can be used to measure cancer pain as well as chronic pain and proved that the Chinese version of BPI (BPI-C) has good internal consistency and acceptable test-retest reliability [32].

### Secondary Outcome

#### Depression Anxiety Stress Scales-21 - Chinese Version

Depression Anxiety Stress Scales-21 (DASS-21) is a self-report instrument to measure three negative emotional states: depression, anxiety, and stress. A higher score indicates a greater level of psychological symptoms. A previous study demonstrated that the Chinese version of DASS-21 has excellent internal consistency and validity [33].

#### Pain Self-Efficacy Questionnaire - Chinese Version

The Pain Self-Efficacy Questionnaire - Chinese Version (PSEQ-C) contains 10 questions regarding a patient’s belief about his or her ability to accomplish the daily tasks despite pain. A higher score reflects stronger pain-related self-efficacy. Internal consistency and validity of the PSEQ-C have been proved [34].

#### Satisfaction, Acceptability, and Learning Performance

Satisfaction and acceptability measures were assessed at the posttreatment assessment. Several questions were asked at the end of the program to assess participants’ satisfaction with and acceptability of the program, such as (1) “Do you think the program is useful?” (2) “Does it worth your time?” and (3) “Would you feel confident to recommend this program?” The questions were used in previous studies to assess the acceptability of the internet-delivered program [19,35]. Open-ended questions were also used, including “How do you think about this program?” and “What are the strengths/disadvantages of the program?” [36] Learning performance was measured at T1 and T2. The score of the MCQs was calculated. A total score of ≥10 was considered a better learning performance.

### Statistical Analysis

The Statistical Package for the Social Sciences (SPSS) version 23 (IBM corporation, Armonk, New York) was used for handling and analyzing the data. The outcome variables and demographic characteristics were presented using descriptive statistics. The differences in demographic characteristics and outcome variables between the two groups were compared using a Chi-square test. Independent sample *t* test was applied to compare the changes in mean scores of the outcome variables. One-way analysis of variance was conducted to test the within-group changes of the outcome at baseline, posttreatment, and 1-month follow-up. Bivariate correlation was used to assess the correlation between the dosage of intervention (ie, frequency of reading the online materials in WeChat) and the outcome variables. The significance level was set at .05 (two tailed); a *P* value <.05 was considered statistically significant. Responses to open-ended questions on satisfaction with and acceptability of this online program were analyzed using a conventional content analysis.

## Results

### Baseline Characteristics

The baseline demographics, pain-related characteristics, and baseline outcome of all the participants are presented in Multimedia Appendix 2. The results suggested that more female participants experienced pain than male participants in both groups. More than half the participants were aged between 21 and 30 years. The study involved a predominantly college-educated population (91/95, 96%). In all, 34.7% of the participants were professionals, and 20% had a monthly salary over 10,000 CNY (US $1488), which accounts for the highest proportion in our study. Most of the participants were living in the Southern and Northwest China. There was no significant difference between any of the baseline characteristics of the two groups.

### Pain: Experimental Group Versus Control Group Over Time

#### Pain Intensity and Pain Interference

As presented in Table 1, the overall mean pain score of the experimental group was significantly lower than that of the control group (*P*=.001). Pain intensity of the experimental group was also significantly different between baseline and posttreatment, while no such difference was observed in the control group. Pain interference improved in the experimental group at T1, and the within-group difference in the experimental group showed statistical significance (*P*<.01). The between-group difference was also statistically significant.

**Table 1:** Pain: Experimental group versus control group over time.

Group (time point)			Experimental group, mean (SD)		Control group, mean (SD)		Mean difference		Cohen *d*^a^ (95% CI)		*P* value^b^
**Pain intensity**											
	Baseline (T0)	4.19 (2.07)^c^		4.02 (2.19)		0.171		0.080 (−0.698 to 1.039)		.70	
	Posttreatment (T1)	3.17 (1.15)^c^		4.26 (1.60)		−1.090		−0.784 (−1.715 to −0.466)		.001	
	One-month follow-up (T2)	3.85 (1.58)		3.58 (2.07)		0.270		0.147 (−0.890 to 1.429)		.64	
**Pain interference**											
	Baseline (T0)	2.75 (1.53)^d^		2.84 (1.40)		–0.086		−0.051 (−0.768 to 0.597)		.80	
	Posttreatment (T1)	2.36 (0.40)^d^		2.98 (0.67)		–0.620		−1.139 (−0.872 to −0.381)		<.001	
	One-month follow-up (T2)	3.11 (1.89)		2.71 (1.10)		0.400		0.255 (−0.772 to 1.559)		.50	
**Pain self-efficacy**											
	Baseline (T0)	43.09 (15.46)		46.38 (14.43)		−3.290		−0.220 (−9.382 to 2.803)		.29	
	Posttreatment (T1)	46.52 (8.83)		45.34 (10.04)		1.181		0.125 (−3.103 to 5.465)		.58	
	One-month follow-up (T2)	46.12 (11.44)		47.25 (11.03)		−1.132		−0.101(−8.806 to 6.541)		.77	

^a^Guideline for Cohen *d*: small, *d*=0.2; medium, *d*=0.5; and large, *d*=0.8.

^b^Independent sample *t* test was applied. A *P* value <.05 was considered statistically significant.

^c^One-way analysis of variance was applied, *P*=.012. Pain intensity at T0 was greater than that at T1.

^d^One-way analysis of variance was applied, *P*<.01. Pain interference at T0 was greater than that at T1.

#### Pain Self-Efficacy

Results of pain self-efficacy questionnaire in the two groups over time are reported in Table 1. A clinical improvement in pain self-efficacy was observed in the experimental group after the program. There were no statistically significant changes in the control group. The between-group differences were not significant over time.

#### Depression, Anxiety, and Stress

**Figure 2:**
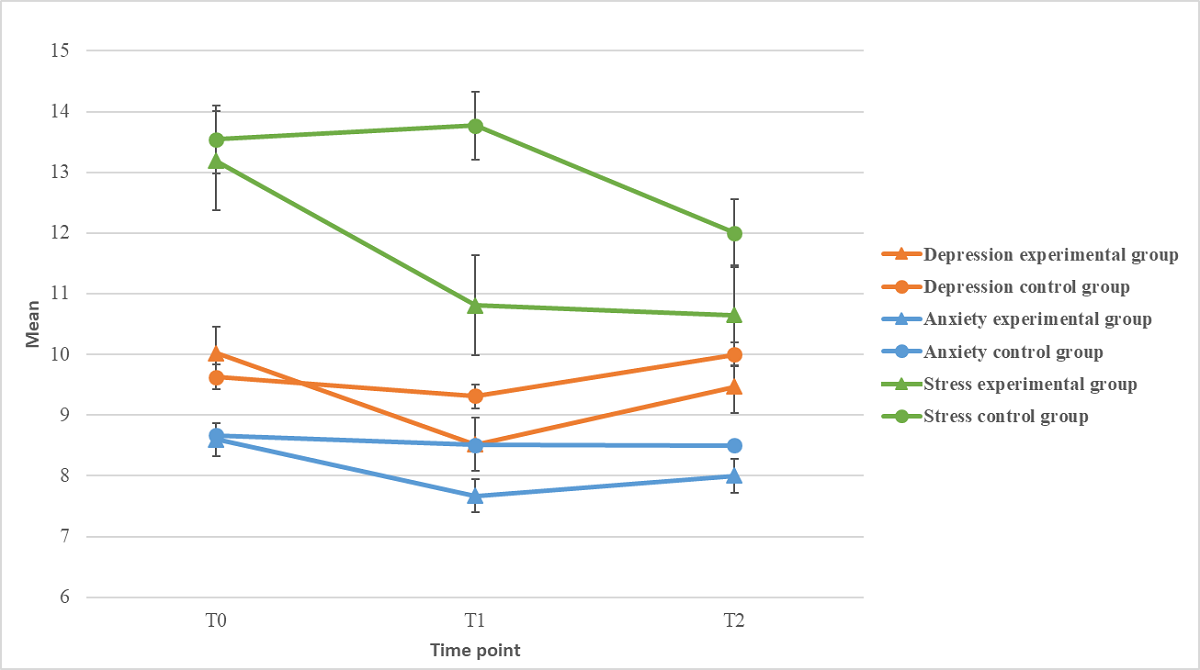
Depression, anxiety, and stress over time.

Changes in depression, anxiety, and stress are shown in Figure 2. Improvements in depression, anxiety, and stress were shown in the experimental group; however, the differences were not significant. Depression and anxiety in the control group reduced slightly, whereas the stress level increased slightly. The between-group differences were nonsignificant.

#### Dosage of Intervention (Frequency of Reading the Online Materials in WeChat)

The average dosage of intervention (ie, frequency of reading the online materials in WeChat) in the education period (ie, T0 to T1) was 4.07 (SD 1.76), which is higher than that in the follow-up period (ie, T1 to T2; 2.68 [1.72]). The difference between the two periods was statistically significant (*P*=.001). The correlation between the dosage of intervention and pain intensity, depression, anxiety, and stress was demonstrated. At the 1-month follow-up, a significant correlation was observed between the dosage of intervention and pain intensity/depression (Table 2).

**Table 2:** Correlation between dosage and outcome variables.

Variable	T1 (posttreatment)		T2 (1-month follow-up)			
	*r* ^a^	*P* value		*r*	*P* value	
Pain intensity	−0.393^b^	.01		−0.599^b^		<.001
Pain interference	0.217	.17		−0.180		.31
Pain self-efficacy	0.081	.61		0.122		.49
Depression	−0.564^b^	<.001		−0.726^b^		<.001
Anxiety	−0.316^c^	.04		−0.070		.70
Stress	−0.310^c^	.05		−0.040		.82

^a^*r* is calculated using the Pearson correlation. Guideline: small, *r*=0.10 to 0.29; medium, *r*=0.30 to 0.49; large, *r*=0.50 to 1.0.

^b^Correlation is significant at .01 level (two-tailed).

^c^Correlation is significant at .05 level (two-tailed).

#### Learning Performance, Satisfaction, and Acceptability of the Online Program

The overall mean score of the MCQs for the experimental group was 9.67 (SD 1.028) of 10. In all, 76% (32/42) participants reported that they were satisfied with this online pain education program, and 69% (29/42) felt it was worth spending time on. Moreover, 33 (78.57%) participants showed willingness to recommend this program to others. Answers for open-ended questions also showed participants’ satisfaction and acceptability: “the program is quite convenient,” “the knowledge is useful,” and “will recommend to others.”

## Discussion

### Principal Findings

This study aimed to evaluate the effectiveness of an online pain education program and participants’ satisfaction and acceptability. After our education program, the pain intensity reduced significantly in the experimental group, and depression, anxiety, stress, and pain self-efficacy showed clinical improvement. A significant correlation was demonstrated between depression, anxiety, and dosage of the intervention. Our results also showed that this online program was acceptable, and participants were satisfied with the program and willing to recommend it to others.

In this study, participants were recruited online via WeChat. Open recruitment through local media or the internet is likely to attract individuals who are more motivated to participate [37]. Consistent with this finding, we found a high completion rate of 89% (42/27) in the experimental group. Results showed that the learning performance of participants was acceptable with the mean score of 9.67/10. This may be because of the socioeconomic status of the participants. Research has indicated that the proportion of people with reliable access to the internet is lower among those with lower socioeconomic status [18,38,39], and a higher risk of pain is correlated with a lower sociodemographic status [11,18,40]. Although the emerging evidence of the internet-delivered pain management program is encouraging, there are still many people who do not have reliable access to the internet and thus have difficulty in utilizing the online programs [18]. As reported by the National Bureau of Statistics of China in 2017, the average monthly income of general Chinese population was 2165 Yuan (US $318) and was lower in the rural areas [41]. The monthly income of approximately 73% of our participants was higher than the average among the general population. The income was high enough to cover the cost of the internet. In addition, participants with a high educational level accounted for the highest proportion. The good learning ability is indicated with the high level of education. Therefore, for most of the working adults in China, sufficient affordability and learning ability make the online pain education program applicable.

Major findings according to the primary outcome measures suggested that this pain education program has the potential to reduce the pain intensity. The result of the significant reduction of pain intensity is consistent with the previous studies conducted among different population in other countries [4,16,18,42]. Studies on internet-delivered pain management programs demonstrated that participants’ symptoms are relatively stable over time among those in the treatment-as-usual control group (without a target intervention) [18,19,36,43].

In our study, the significant reduction in pain interference indicated that participants had become more functional and could self-manage their pain more effectively in the daily life [44]. In the experimental group, compared with the control group, the reduction in pain interference was significant, indicating that our online pain education program has the potential to reduce disturbances caused by pain. Our results are consistent with those of previous online pain management studies that reported improved pain interference after the intervention [44-46].

However, the changes in pain intensity and pain interference were statistically nonsignificant in the control group, and the differences in the 1-month follow-up in this study were not significant between the two groups. This may relate to the loss to follow-up rate. As noted in this study, a rate higher than 50% (23/35) was demonstrated in the control group, which is considered one of the reasons for the nonsignificant differences within the control group over time. In addition, the one-page simple material provided at beginning to the control group perhaps was insufficient, and no interaction with participants may impact the outcome as well.

Only clinical improvements were observed in depression, anxiety, stress, and self-efficacy because of the length of the intervention and the follow-up period; a period of 1 month is possibly too short to achieve a significant effect. Contrary to the expectations, the stress level of the control group increased at the posttreatment assessment. This unexpected result may be because of the factors not related to the education program, such as participants’ heavy and busy work, as the participants were working adults. Other stress from their daily life rather than pain-related stress may also have impacted the outcome.

It is noteworthy that a significant correlation between the dosage of the intervention and the outcome variables was demonstrated in our study. In a pain self-management study, Nicholas et al [15] reported that a higher dose of the intervention could partially explain the better outcome achieved, which was consistent with our findings that a higher dosage of intervention resulted in less pain and better pain-related emotional well-being. However, the previous study provided the intervention using a face-to-face approach. Our results proved that dosage was an important factor that impacts the outcome in the internet-delivered program, which is similar to the face-to-face program.

The higher dosage of intervention in the education period compared with the follow-up is probably because of the reminders we sent. The reminders were sent regularly on a weekly basis, with the purpose of encouraging and supporting the participants to work through the education program and keep reading the materials. Although we expected the participants to read the materials and learn consciously, sometimes, participants may forget to do so when they are busy with work. A regular reminder is a good way to enhance participation. In the previous online pain management programs, emails or phone calls were used to prompt individuals to learn the information and apply the skills taught, and they also helped decrease the dropout rate and facilitate compliance [4,18,36,47]. An internet-based intervention study successfully used such email prompts to encourage use and return visits to online resources [48]. In addition, it was demonstrated that such prompts do not compromise the clinical outcomes and acceptability of the program [49]. WeChat massages were used as an alternative in our study and were effective in enhancing participation.

A previous online pain management program stated it did not involve any interactive component [19]; thus, the interactive module designed in our study was unique. Participants’ understanding of the knowledge provided can be improved during the interaction. The MCQs designed after each article can encourage participants to apply the knowledge learned immediately. Involving some interaction in an education program, particularly for the online program, is necessary. A previous study highlighted the importance of interaction in high-quality education [50]; the researchers stated concluded that the interaction is the most fundamental form of the education. In addition, increasing the amount of interaction can lead to more effective learning and improve satisfaction with the education program as well as the learning outcome [51]. Wright [52] illustrated that if the intervention includes a forum where participants can interact and support each other, they can gain some benefits from similar experiences of other participants. The interactive component including the MCQs and WeChat group designed in this study played a crucial role.

Our study has a number of strengths. First, this study was the first randomized controlled trial designed to explore an education program using an online approach for working adults to self-manage pain in China. Second, a high completion rate of the education program and questionnaires was achieved. Third, an interactive component was involved in this online program, and participants’ learning performance was evaluated. Fourth, we explored the correlation between the dosage of intervention and outcome. Finally, we supplemented satisfaction and acceptability ratings with qualitative analysis of participants’ feedback.

### Limitations

There are several limitations to this study. First, the sample size of the study was relatively small, which limited the statistical power to detect smaller effects. It is possible that significant differences would have been observed with a large sample size. In addition, the 1-month follow-up of participants in our study may not be sufficient. Indeed, a long-term follow-up (eg, ≥6 months) would assist in observing the long-term effect of the program [36]. The completion rate of 48.4% in the 1-month follow-up may indicate the need to have more frequent reminders, to retain the participants in the program in future studies.

### Conclusions

Our findings highlight the significant potential of this online education program in the treatment of pain. Pain intensity reduced significantly after the education program, and pain-related emotional well-being was found to clinically improve. A significant correlation was demonstrated between depression, anxiety, and dosage of the intervention. We conclude that this online program is acceptable. Further promotion to the public can be made to help more people with pain.

## Appendix

Multimedia Appendix 1 Content of the pain education program.

Multimedia Appendix 2 Baseline characteristics of participants.

Multimedia Appendix 3 CONSORT-EHEALTH checklist (V 1.6.1).

## Abbreviations

BPI: Brief Pain Inventory
CVI: Content Validity Index
DASS-21: Depression Anxiety Stress Scales-21
MCQ: multiple choice question
PSEQ-C: Pain Self-Efficacy Questionnaire-Chinese Version

## References

[1] Hayes S, Hogan M, Dowd H, Doherty E, O'Higgins S, Gabhainn SN, MacNeela P, Murphy AW, Kropmans T, O'Neill C, Newell J, McGuire BE (2014). Comparing the clinical-effectiveness and cost-effectiveness of an internet-delivered Acceptance and Commitment Therapy (ACT) intervention with a waiting list control among adults with chronic pain: study protocol for a randomised controlled trial. BMJ Open.

[2] Peng WL, Wu GJ, Sun WZ, Chen JC, Huang AT (2006). Multidisciplinary management of cancer pain: a longitudinal retrospective study on a cohort of end-stage cancer patients. J Pain Symptom Manage.

[3] Reyes-Gibby CC, Duc NB, Yen NP, Nga NH, Tran TV, Guo H, Bhat S, Cleeland C (2006). Status of cancer pain in Hanoi, Vietnam: a hospital-wide survey in a tertiary cancer treatment center. J Pain Symptom Manage.

[4] Berman RL, Iris MA, Bode R, Drengenberg C (2009). The effectiveness of an online mind-body intervention for older adults with chronic pain. J Pain.

[5] Morone NE, Greco CM, Weiner DK (2008). Mindfulness meditation for the treatment of chronic low back pain in older adults: a randomized controlled pilot study. Pain.

[6] (2016). Ausmed.

[7] de Vlieger P, Crombez G, Eccleston C (2006). Worrying about chronic pain. An examination of worry and problem solving in adults who identify as chronic pain sufferers. Pain.

[8] Hanssen DJ, Naarding P, Collard RM, Comijs HC, Voshaar RC (2014). Physical, lifestyle, psychological, and social determinants of pain intensity, pain disability, and the number of pain locations in depressed older adults. Pain.

[9] Tsang A, von Korff M, Lee S, Alonso J, Karam E, Angermeyer MC, Borges GL, Bromet EJ, Demytteneare K, de Girolamo G, de Graaf R, Gureje O, Lepine J, Haro JM, Levinson D, Browne MA, Posada-Villa J, Seedat S, Watanabe M (2008). Common chronic pain conditions in developed and developing countries: gender and age differences and comorbidity with depression-anxiety disorders. J Pain.

[10] Mohamed Zaki LR, Hairi NN (2015). A systematic review of the prevalence and measurement of chronic pain in Asian adults. Pain Manag Nurs.

[11] Chen B, Li L, Donovan C, Gao Y, Ali G, Jiang Y, Xu T, Shan G, Sun W (2016). Prevalence and characteristics of chronic body pain in China: a national study. Springerplus.

[12] Jackson T, Chen H, Iezzi T, Yee M, Chen F (2014). Prevalence and correlates of chronic pain in a random population study of adults in Chongqing, China. Clin J Pain.

[13] Li Y, Tse MM (2019). Pain situations among working adults and the educational needs identified: an exploratory survey via WeChat. BMC Public Health.

[14] Ariëns GA, van Mechelen W, Bongers P, Bouter L, van der Wal G (2001). Psychosocial risk factors for neck pain: a systematic review. Am J Ind Med.

[15] Nicholas MK, Asghari A, Blyth FM, Wood BM, Murray R, McCabe R, Brnabic A, Beeston L, Corbett M, Sherrington C, Overton S (2013). Self-management intervention for chronic pain in older adults: a randomised controlled trial. Pain.

[16] Cosio D, Lin EH (2013). Effects of a pain education program for veterans with chronic, noncancer pain: a pilot study. J Pain Palliat Care Pharmacother.

[17] Chou PL, Lin CC (2011). A pain education programme to improve patient satisfaction with cancer pain management: a randomised control trial. J Clin Nurs.

[18] Dear BF, Gandy M, Karin E, Ricciardi T, Fogliati VJ, McDonald S, Staples LG, Perry KN, Sharpe L, Nicholas MK, Titov N (2017). The pain course: a randomised controlled trial comparing a remote-delivered chronic pain management program when provided in online and workbook formats. Pain.

[19] Dear BF, Gandy M, Karin E, Staples LG, Johnston L, Fogliati VJ, Wootton BM, Terides MD, Kayrouz R, Perry KN, Sharpe L, Nicholas MK, Titov N (2015). The Pain Course: a randomised controlled trial examining an internet-delivered pain management program when provided with different levels of clinician support. Pain.

[20] Gerber BS (2006). The chronic disease self-management program: extending reach through the internet. Med Care.

[21] Keogh E, Rosser BA, Eccleston C (2010). e-Health and chronic pain management: current status and developments. Pain.

[22] Tse MM, Tang A, Budnick A, Ng SS, Yeung SS (2017). Pain and pain management among university students: online survey and web-based education. Cyberpsychol Behav Soc Netw.

[23] CNNIC.

[24] (2017). WeChat Blog: Chatterbox.

[25] Zheng B, Zhao K (2015). Yong Hu Shi Yong Wei Xin Ding Yue Hao de Yuan Yin Tan Jiu. West Journal.

[26] WeChat Help Center.

[27] Yeh M, Chen H, Houng Y, Liao F (2006). A New Websitelf-Management of Dysmenorrhea Online Learning. Beijing Biomedical Engineering.

[28] Random.org - True Random Number Service.

[29] Koo TK, Li MY (2016). A guideline of selecting and reporting intraclass correlation coefficients for reliability research. J Chiropr Med.

[30] Koo TK, Li MY (2016). A guideline of selecting and reporting intraclass correlation coefficients for reliability research. J Chiropr Med.

[31] Li B (2018). Eastern Theater General Hospital – Neurology.

[32] Wang XS, Mendoza TR, Gao SZ, Cleeland CS (1996). The Chinese version of the Brief Pain Inventory (BPI-C): its development and use in a study of cancer pain. Pain.

[33] Wen Y, Wu DX, Lv X-J, Li HG, Liu XC, Yang YP, XU YX, Zhao Y (2012). Psychometric properties of the Chinese Short Version of Depression Anxiety and Stress Scale in Chinese adults. Chinese J Public Heal.

[34] Lim HS, Chen PP, Wong TC, Gin T, Wong E, Chan IS, Chu J (2007). Validation of the Chinese version of pain self-efficacy questionnaire. Anesth Analg.

[35] Titov N, Dear BF, Ali S, Zou JB, Lorian CN, Johnston L, Terides MD, Kayrouz R, Klein B, Gandy M, Fogliati VJ (2015). Clinical and cost-effectiveness of therapist-guided internet-delivered cognitive behavior therapy for older adults with symptoms of depression: a randomized controlled trial. Behav Ther.

[36] Friesen LN, Hadjistavropoulos HD, Schneider LH, Alberts NM, Titov N, Dear BF (2017). Examination of an internet-delivered cognitive behavioural pain management course for adults with fibromyalgia: a randomized controlled trial. Pain.

[37] Bender JL, Radhakrishnan A, Diorio C, Englesakis M, Jadad AR (2011). Can pain be managed through the Internet? A systematic review of randomized controlled trials. Pain.

[38] Wang JY, Bennett K, Probst J (2011). Subdividing the digital divide: differences in internet access and use among rural residents with medical limitations. J Med Internet Res.

[39] Ennis L, Rose D, Denis M, Pandit N, Wykes T (2012). Can't surf, won't surf: the digital divide in mental health. J Ment Health.

[40] Miljkovic A, Stipcic A, Braš M, Dorđević V, Brajkovic L, Hayward C, Pavic A, Kolcic I, Polašek O (2014). Is experimentally induced pain associated with socioeconomic status? Do poor people hurt more?. Med Sci Monit.

[41] National BOSOC National Bureau of Statistics of China.

[42] Ruehlman LS, Karoly P, Enders C (2012). A randomized controlled evaluation of an online chronic pain self management program. Pain.

[43] Dear BF, Titov N, Perry KN, Johnston L, Wootton BM, Terides MD, Rapee RM, Hudson JL (2013). The Pain Course: a randomised controlled trial of a clinician-guided internet-delivered cognitive behaviour therapy program for managing chronic pain and emotional well-being. Pain.

[44] Dysvik E, Kvaløy JT, Stokkeland R, Natvig GK (2010). The effectiveness of a multidisciplinary pain management programme managing chronic pain on pain perceptions, health-related quality of life and stages of change--A non-randomized controlled study. Int J Nurs Stud.

[45] Brattberg G (2006). Internet-based rehabilitation for individuals with chronic pain and burnout: a randomized trial. Int J Rehabil Res.

[46] Devineni T, Blanchard EB (2005). A randomized controlled trial of an internet-based treatment for chronic headache. Behav Res Ther.

[47] Andersson G, Lundström P, Ström L (2003). Internet-based treatment of headache: does telephone contact add anything?. Headache.

[48] McNeill LH, Viswanath K, Bennett GG, Puleo E, Emmons KM (2007). Feasibility of using a web-based nutrition intervention among residents of multiethnic working-class neighborhoods. Prev Chronic Dis.

[49] Titov N, Andrews G, Davies M, McIntyre K, Robinson E, Solley K (2010). Internet treatment for depression: a randomized controlled trial comparing clinician vs. technician assistance. PLoS One.

[50] Su B, Bonk C, Magjuka R, Liu X, Lee S (2005). The importance of interaction in web-based education: a program-level case study of online MBA courses. J Interact Online Learn.

[51] Zhang S (1994). Are interaction time and psychological interactivity the same thing in the distance learning television classroom?. Educ Technol.

[52] Wright K (2002). Social support within an on-line cancer community: an assessment of emotional support, perceptions of advantages and disadvantages, and motives for using the community from a communication perspective. J Appl Commun Res.

