# Supplementary material for: An Online Pain Education Program for Working Adults: Pilot Randomized Controlled Trial
Source: J Med Internet Res. 2020 Jan 14;22(1):e15071. doi: 10.2196/15071 (PMC6996734; doi:10.2196/15071)
Supplement: Multimedia Appendix 1 [file jmir_v22i1e15071_app1.docx]

| No. | Topic | Details | | Interactive component |
| --- | --- | --- | --- | --- |
| 1 | Basic knowledge of pain | Definition and symptoms of pain | | (1) MCQs^a^: (a) 3-4 MCQs related to the topic were asked after each lesson. (b) Participants were given unlimited attempts to complete the questions. (c) The total score of each lesson was 10, and participants’ score was recorded to evaluate the learning performance. (d) Correct answer was provided automatically after completing the MCQs. (2) A WeChat Group was created for questions and answers. (3) Regular reminders were sent to the participants weekly to remind them to continue reading our materials. |
|  |  | Pathogeny of pain | |  |
|  |  | Relationship with disease | |  |
|  |  | Physical and psychological impact of pain | |  |
| 2 | Pharmacy treatment | Opioid analgesics | Effectiveness;  usage;  side-effect |  |
|  |  | Nonopioid analgesics |  |  |
|  |  | Adjunctive analgesics |  |  |
| 3 | Nonpharmacy treatment | Exercise and posture | |  |
|  |  | Nutrition | |  |
|  |  | Traditional Chinese medication | Acupuncture; cupping;  acupressure |  |
|  |  | Complementary choices | Breathing; aromatherapy; heat and cold; music; laughter; relaxation |  |
| 4 | Relevant resources | Sleep management; mood management (anxiety and depression) | |  |

Table 1. Content of the pain education program.

^a^MCQs: multiple choice questions.
