# Supplementary material for: An Online Pain Education Program for Working Adults: Pilot Randomized Controlled Trial
Source: J Med Internet Res. 2020 Jan 14;22(1):e15071. doi: 10.2196/15071 (PMC6996734; doi:10.2196/15071)
Supplement: Multimedia Appendix 2 [file jmir_v22i1e15071_app2.docx]

Table 2. Baseline characteristics of participants.

| Variables | | Total (N=95) | Experimental group (n=47) | Control group (n=48) | *P* value^a^ |
| --- | --- | --- | --- | --- | --- |
| **Gender, n (%)** | | — | — | — | .127 |
|  | Female | 68 (71.6) | 37 (78.7) | 31 (64.6) | — |
|  | Male | 27 (28.4) | 10 (21.3) | 17 (35.4) | — |
| **Age (years), n (%)** | | — | — | — | .06 |
|  | 16-20 | 1 (1.1) | 0 | 1 (2.1) | — |
|  | 21-30 | 53 (55.8) | 25 (53.2) | 28 (58.3) | — |
|  | 31-40 | 11 (11.6) | 9 (19.1) | 2 (4.2) | — |
|  | 41-50 | 19 (20.0) | 6 (12.8) | 13 (27.1) | — |
|  | 51-60 | 11 (11.6) | 7 (14.9) | 4 (8.3) | — |
| **Marital status, n (%)** | | — | — | — | .277 |
|  | Married | 47 (49.5) | 26 (55.3) | 21 (43.8) | — |
|  | Single | 47 (49.5) | 20 (42.6) | 27 (56.3) | — |
|  | Divorced/widowed | 1 (1.1) | 1 (2.1) | 0 | — |
| **Education level, n (%)** | | — | — | — | .98 |
|  | College or above | 91 (95.8) | 45 (95.7) | 46 (95.8) | — |
|  | High school/less | 4 (4.2) | 2 (4.3) | 2 (4.2) | — |
| **Occupation, n (%)** | | — | — | — | .53 |
|  | Profession | 33 (34.7) | 18 (38.3) | 15 (31.3) | — |
|  | Others | 27 (28.4) | 15 (31.9) | 12 (25.0) | — |
|  | Business/service | 13 (13.7) | 4 (8.5) | 9 (18.8) | — |
|  | Worker | 10 (10.5) | 4 (8.5) | 6 (12.5) | — |
|  | Civil | 7 (7.4) | 4 (8.5) | 3 (6.3) | — |
|  | Farmer | 1 (1.1) | 1 (2.1) | 0 | — |
|  | Produce/transport | 2 (2.1) | 1 (2.1) | 1 (2.1) | — |
|  | Soldier | 2 (2.1) | 0 | 2 (4.2) | — |
| **Living condition, n (%)** | | — | — | — | .88 |
|  | With mate | 22 (23.2) | 12 (25.5) | 10 (20.8) | — |
|  | With mate and children | 21 (22.1) | 10 (21.3) | 11 (22.9) | — |
|  | With parents | 18 (18.9) | 8 (17.0) | 10 (20.8) | — |
|  | With friends | 17 (17.9) | 8 (17.0) | 9 (18.8) | — |
|  | Along | 16 (16.8) | 9 (19.1) | 7 (14.6) | — |
|  | With children | 1 (1.1) | 0 | 1 (2.1) | — |
| **Income (CNY/month, 1 CNY≈US $0.15), n (%)** | | — | — | — | .051 |
|  | <2000 | 14 (14.7) | 7 (14.9) | 7 (14.6) | — |
|  | 2001-3000 | 4 (4.2) | 2 (4.3) | 2 (4.2) | — |
|  | 3001-4000 | 8 (8.4) | 4 (8.5) | 4 (8.3) | — |
|  | 4001-5000 | 10 (10.5) | 5 (10.6) | 5 (10.4) | — |
|  | 5001-6000 | 10 (10.5) | 5 (10.6) | 5 (10.4) | — |
|  | 6001-7000 | 8 (8.4) | 5 (10.6) | 3 (6.3) | — |
|  | 7001-8000 | 12 (12.6) | 10 (21.3) | 2 (4.2) | — |
|  | 8001-9000 | 5 (5.3) | 0 | 5 (10.4) | — |
|  | 9001-10,000 | 5 (5.3) | 4 (8.5) | 1 (2.1) | — |
|  | >10,000 | 19 (20.0) | 5 (10.6) | 14 (29.2) | — |
| **Living place in China, n (%)** | | — | — | — | .567 |
|  | Southern | 36 (37.9) | 18 (38.3) | 18 (37.5) | — |
|  | Northwest | 33 (34.7) | 17 (36.2) | 16 (33.3) | — |
|  | Eastern | 10 (10.5) | 3 (6.4) | 7 (14.6) | — |
|  | Northern | 6 (6.3) | 4 (8.5) | 2 (4.2) | — |
|  | Northeast | 5 (5.3) | 2 (4.3) | 3 (6.3) | — |
|  | Central | 3 (3.2) | 1 (2.1) | 2 (4.2) | — |
|  | Southwest | 2 (2.1) | 2 (4.3) | 0 | — |
| **Pain measures, mean (SD)** | | | | | |
|  | Brief Pain Inventory-sf worst pain^b^ | 4.11 (2.12) | 4.19 (2.07) | 4.02 (2.19) | .846 |
|  | Brief Pain Inventory-sf pain interference^c^ | 2.79 (2.30) | 2.95 (2.35) | 2.65 (2.25) | .097 |
| **Psychological assessment, mean (SD)** | | | | | |
|  | Depression, Anxiety, and Stress Scale depression subscale^d^ | 9.82 (11.00) | 10.02 (10.59) | 9.63 (11.49) | .571 |
|  | Depression, Anxiety, and Stress Scale anxiety subscale^e^ | 8.63 (8.86) | 8.60 (7.49) | 8.67 (10.09) | .377 |
|  | Depression, Anxiety, and Stress Scale stress subscale^f^ | 13.37 (11.23) | 13.19 (11.23) | 13.54 (11.35) | .664 |
| Pain self-efficacy^g^, mean (SD) | | 44.75 (14.96) | 43.09 (15.46) | 46.38 (14.43) | .181 |

^a^Chi-square test was applied, and a *P* value <.05 is considered statistically significant.

^b^11-point Likert scale: 0=no pain; 10=the worst pain.

^c^11-point Likert scale: 0=does not interfere; 10=completely interfere.

^d^Depression score on Depression, Anxiety, and Stress Scale (DASS-21) ranges from 0 to 3 for each item, 0=do not apply to me at all, 3=apply to me very much or most of the time. A higher total score indicates a greater degree of depression.

^e^Anxiety score on Depression, Anxiety, and Stress Scale (DASS-21) ranges from 0 to 3 for each item, 0=do not apply to me at all, 3=apply to me very much or most of the time.

^f^Stress score on Depression, Anxiety, and Stress Scale (DASS-21) ranges from 0 to 3 for each item, 0=do not apply to me at all, 3=apply to me very much or most of the time.

^g^Pain Self-Efficacy Questionnaire was used; 7-point Likert scale for each item, 0=not at all confident, 7=completely confident.
